# Objective Assessments of Mental Fatigue During a Continuous Long-Term Stress Condition

**DOI:** 10.3389/fnhum.2021.733426

**Published:** 2021-11-10

**Authors:** Han Zhang, Jingying Wang, Xinyi Geng, Chuantao Li, Shouyan Wang

**Affiliations:** ^1^Institute of Science and Technology for Brain-Inspired Intelligence, Fudan University, Shanghai, China; ^2^School of Optometry, The Hong Kong Polytechnic University, Kowloon, Hong Kong SAR, China; ^3^Naval Medical Center of PLA, Department of Aviation Medicine, Naval Military Medical University, Shanghai, China; ^4^Key Laboratory of Computational Neuroscience and Brain-Inspired Intelligence (Fudan University), Ministry of Education, Shanghai, China; ^5^MOE Frontiers Center for Brain Science, Fudan University, Shanghai, China; ^6^Zhangjiang Fudan International Innovation Center, Shanghai, China; ^7^Shanghai Engineering Research Center of AI and Robotics, Fudan University, Shanghai, China; ^8^Engineering Research Center of AI and Robotics, Ministry of Education, Fudan University, Shanghai, China

**Keywords:** long-term, high-level stress, mental fatigue, EEG, Rényi wavelet entropy, competition

## Abstract

Prolonged periods of cognitive workload will cause mental fatigue, but objective, quantitative, and sensitive measurements that reflect long-term, stress-induced mental fatigue have yet to be elucidated. This study aims to apply a potential marker of Rényi entropy to investigate the mental fatigue changes in a long-term, high-level stress condition and compare three different instruments for assessment of mental fatigue: EEG, the oddball task, and self-scoring. We recruited nine individuals who participated in a 5-day intellectually challenging competition. The participants were assessed for mental fatigue each day of the competition using prefrontal cortex electroencephalogram (EEG). Reaction time in an oddball task and self-rated scoring were used comparatively to evaluate the performance of the EEG. Repeated measures ANOVA was utilized to analyze the differences among score, reaction time, and wavelet Rényi entropy. The results demonstrated that both wavelet Rényi entropy extracted from EEG and self-rated scoring revealed significant increases in mental fatigue during the 5 days of competition (*P* < 0.001). The reaction time of the oddball task did not show significant changes during the five-day competition (*P* = 0.066). Moreover, the wavelet Rényi entropy analysis of EEG showed greater sensitivity than the self-rated scoring and reaction time of the oddball task for measuring mental fatigue changes. In conclusion, this study shows that mental fatigue accumulates during long-term, high-level stress situations. The study also indicates that EEG wavelet Rényi entropy is an efficient metric to reflect the change of mental fatigue under a long-term stress condition and that EEG is a better method to assess long-term mental fatigue.

## Introduction

Mental fatigue refers to the feeling experienced after or during prolonged periods of cognitive activity and has been associated with a temporary inability to maintain optimal cognitive performance (Borghini et al., 2014). Increased mental effort can induce mental fatigue. In short-term, it can impair vigilance, reaction time, and physical performance, reducing work capacity. This is particularly true in real-world situations, where some jobs require sustained concentration, such as police officer, medical worker, and pilot. These occupations often endure long work hours with high stress, increasing the risk of accidents that lead to injury and substantial economic loss (Ricci et al., 2007; Fekedulegn et al., 2018; Jensen, 2018; Dohrmann et al., 2019; De Jong et al., 2020). Moreover, people in a state of constant fatigue over a long period have a higher risk of morbidity (De Vries et al., 2015). Therefore, it is crucial to measure mental fatigue in people whose work requires high levels of mental effort to prevent health problems and subsequent economic loss.

As mental fatigue receives increasing attention, researchers have come up with many methods to quantify the severity of the mental fatigue state. Self-rating scales are a widely used method, such as the Stanford sleepiness scale (Duan et al., 2018), and the Karolinska sleepiness scale (Liu J. et al., 2010). Additionally, many scales were designed to fit specific situations, such as the Fatigue Severity Scale (Loy et al., 2018) and the Swedish Occupational Fatigue Inventory (Hendrawan et al., 2018). Other frequently used instruments are cognitive tasks (e.g., the psychomotor vigilance task, and the oddball task) which were used to evaluate changes in fatigue-related cognitive components (Dinges et al., 1997; Ciria et al., 2017; Phillips et al., 2017; Dimitrakopoulos et al., 2018; Lin et al., 2018; Qi et al., 2019). Physiological measures that relate to mental fatigue, such as eye blink rate, heart rate variability, or brain activity through electroencephalogram (EEG) and dynamic functional connectivity (Kar et al., 2010; Borghini et al., 2013; Keshmiri et al., 2019; Foong et al., 2020; Qi et al., 2020) are also frequently investigated in researche— especially in brain activity research, as they can provide a direct measure of brain status transformation during the mental fatigue process.

Because of the difficulty of obtaining real-world recordings, research on mental fatigue has been chiefly done using self-rating questionnaires or scales. Although self-report scales are convenient and widely used, the results are influenced by subjective bias (Boksem and Tops, 2008); however, objective quantitative methods like the cognitive tasks mentioned above are less suitable for real-time detection while doing other task. The advantages of monitoring physiological features are that participants will not be distracted by the measurement procedures, researchers can observe the mental fatigue generating process, and there is no learning effect.

In recent years, accumulated research shows that EEG band alterations are highly correlated with mental fatigue, suggesting that EEG has excellent potential for measuring mental fatigue (Han et al., 2019; Qi et al., 2019; Sikander and Anwar, 2019; Jing et al., 2020). However, these studies were focused on vehicle driving, and conduct in experimental conditions (Dimitrakopoulos et al., 2018). Less is known about EEG signal changes during mental fatigue generated by long-term high-level stress conditions, even though it is common in certain occupations.

In this study, we aim to apply a potential marker of Rényi entropy to investigate the change of mental fatigue during a long-term stress condition by measuring EEG over the prefrontal cortex (PFC). We measured mental fatigue each day for 5 days during a real-life intellectually challenging competition and performed a wavelet Rényi entropy analysis (Rosso et al., 2006) of the EEG to estimate the fatigue changes. Moreover, we evaluated the performance of the EEG analyses by comparing with different measurement methods: self-rating scale and reaction time during a visual and audio oddball task. We hypothesized that the mental fatigue would accumulate throughout the 5 days continuous stress condition, and that the analysis of PFC EEG wavelet Rényi entropy would be able to distinguish the mental fatigue status.

## Materials and Methods

### Participants

Inclusion criteria included: good general health, free of neurological diseases and psychiatric disorders, and no history of hearing and visual impairment. Nine volunteers (9 males, age 19–22, mean = 20.2, and SD = 0.79) who attended the National Undergraduate Electronics Design Contest were recruited from the University of Shanghai for Science and Technology (USST). All participants provided written informed consent to the experimental procedures before the experiment. The study was approved by the ethics committee of the institute of science and technology for brain-inspired intelligence (AF/SC-04/20200911). During the experiment, participants were not allowed to consume any form of alcohol, caffeine, or nicotine products.

### Study Design and Procedures

#### Study Design

Participants took part in a 5-day electronics design competition, during which three subjective and objective measurement tools were used to measure their mental fatigue.

The National Undergraduate Electronic Design Contest is a biennial contest where the undergraduates who contend must complete a project within 5 days. Getting an excellent ranking in the competition is beneficial to job searching and master’s applications (Guo and Zhang, 2010). The importance of performing well results in a high level of competition, in which students will be under significant pressure, resulting in the accumulation of mental fatigue.

**FIGURE 1:**
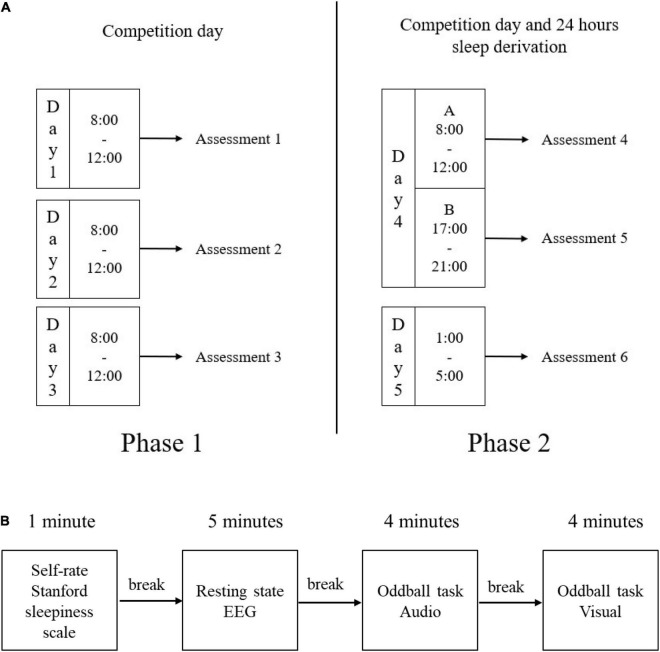
Experimental procedures. **(A)** The experiment included two phases, i.e., phase 1 with each assessment session for the first three consecutive competition days, and phase 2 with three assessment sessions with a 24-h period of sleep deprivation during the last two competition days. **(B)** The assessment included four sessions, which were 1 min self-rating, 5 min resting-state EEG, 4 min audio stimulation oddball task, and 4 min visual simulation oddball task; there were short breaks between each session. EEG = electroencephalography.

To investigate changes in the participants’ mental fatigue during the competition, we performed multiple daily measurements. To capture the changes over time, we divided the assessments into two phases (see Figure 1). The first phase was assessed from day 1 – 3, and the second phase was assessed from day 4-A – 5 (day 4-A was the first assessment in day four). Participants were given adaptation training the day before the start of the experiment on day 0. Further, they were assessed at a fixed time each day in numbered order, thus eliminating the variation due to different assessment times. All participants were assessed once a day during the period from 8 a.m. to 12 p.m. On the 4th day, the competition entered the final stage, and since all participants had given up sleep, two additional assessment rounds were given to all participants. Assessment day 4-B (day 4-B was the second assessment in day four) starting at 5 p.m., and assessment day 5 started the next day at 1 a.m. Assessment Day 5 was completed under sleep deprivation as this was a competition condition. For the first three days of the competition, participants were prohibited from sleeping except for their bedtime. On the phase two of the competition, sleep was prohibited entirely.

#### Procedure

Participants were instructed to familiarize themselves with the oddball task a day before the experiment began to reduce the experimental bias caused by the learning process. A room 10 m away from the competition site was set up with adequate sound and light insulation to conduct experiments. Both the LED light and loudspeaker were 1 meter away from the participant. The experiment was conducted at the University of Shanghai for Science and Technology, and the entire experiment was video recorded.

The experimental procedures for each assessment are illustrated in Figure 1B: first of all, participants filled out the Stanford Sleepiness Scale and reported their sleep duration before the assessment. Then we helped them wear the device and dim the lights in the room. Following was the EEG recording – 5 min resting-state prefrontal cortex (PFC) EEG with closed eyes. Participants were asked to sit in a chair, keep quiet, keep their mood calm, and keep a blank mind. Finally, the oddball task included 4 min of audio and visual stimulation. Participants were asked to be as still as they can except to press the button.

### Physiological Recording

Our team developed a study device that can acquire EEG signals during the performance of the oddball task. The device adopted a high-performance 24-bit analog-to-digital converter ADS1299 chip (chip ADS1299, Texas Instruments, Dallas, TX, United States). It can simultaneously record 8-channel EEG, generate visual and audio stimulation, and capture button input. The data was uploaded in real-time to the computer through Bluetooth wireless communication and backed up on an SD card.

EEG data were collected from four locations (AFp7, Fp1h, Fp2h, and AFp8 according to the 10–5 system [Jurcak et al., 2007]) chosen to cover the prefrontal brain region, and EEG signal amplified 12 times. The reference electrode was placed at the mastoid bone. Our reason for monitoring the EEG signal in the PFC region was two-fold. First, the EEG signal characteristics in the PFC region are different between awake and fatigued states, and these characteristics are suitable for assessing changes in fatigue state (Liu J.P. et al., 2010; Li et al., 2012, 2016; Zou et al., 2020). Second, in practice, the advantage of measuring the non-hair-bearing scalp area in PFC is that the experiment is easy to prepare and is less invasive. If the experiment adopts the standard electrode montage, potential participants may be more likely to refuse to participate in the experiment.

This study analyzed EEG frequency domain characteristics to explore mental fatigue changes (Kong et al., 2017; Van Cutsem et al., 2017). The sampling rate in this experiment was 250 Hz, and the resolution of the EEG signal was two microvolts. The impedance level was ensured to be below 5 kΩ before processing.

### Fatigue Assessment

#### Stanford Sleepiness Scale

The Stanford Sleepiness Scale (SSS) (Hoddes et al., 1972) is a frequently used mental fatigue self-report scale (Duan et al., 2018). The scale has a single item with a scale range from one (“Feeling active and vital; alert; wide awake.”) to seven (“Almost in reverie; sleep onset soon; lost struggle to remain awake.”).

#### Oddball Task

The oddball task was utilized to reflect participants’ mental fatigue by allowing us to investigate the decrease in vigilance when mental fatigue was present (Clayton et al., 2015). In an oddball task, stimuli are presented in a continuous stream, and participants must detect the presence of an oddball stimulus. In this study, there were two kinds of stimuli. One stimulus with a high probability (80%) called standard stimuli, and another with a small probability (20%) is called deviant stimuli. Studies have shown that people’s attention-related responses are different when doing visual and auditory stimuli oddball tasks (Kiat, 2018). Reaction times measured by these two oddball tasks may differ when individuals develop mental fatigue, so two methods were chosen for this study. Participants were asked to respond to the deviant stimuli by using one finger of their dominant hand to press a button as fast as possible. The reaction was determined as the time difference between the onset of the stimuli and the onset of the button pressing.

Two sensory stimuli (visual and auditory) were applied in this study. Two sounds, 1 kHz (standard stimulus) and 2 kHz (deviant stimulus) were played randomly in the audio stimulation paradigm. The interval between sounds was 1000 ms, and the duration was 50 ms. In the visual stimulation paradigm, the background of the standard oddball sequence was gray. The target stimulus was a red light, and the non-target stimulus was a green light. The signal duration was 50 ms, with an interval of 1000 ms. We use participants’ mean reaction time (RT) in each task as metrics.

### Signal Processing

Wavelet transform can be used to extract information from EEG data, such as trends, discontinuities, and repeated patterns (Yildiz et al., 2009). The entropy is used to quantify the amount of uncertainty or randomness in the pattern (Zhang et al., 2014). Entropy based on Shannon’s entropy theory belongs to a short-range or extensive concept (Jiao et al., 2012; Wang et al., 2015). However, biomedical systems are often characterized by long-range interactions or long-term memories. Rényi entropy is a generalized form of entropy, which has advantages in analyzing long-range features (Liang et al., 2015). Rényi entropy differs from Spectral entropy in that the sum is weighted toward frequencies in the lower frequency band. In higher frequency band (20–45 Hz), there are no differences (Guarascio and Puthusserypady, 2017); therefore, we used the wavelet Rényi entropy to explore the mental fatigue characteristics in 5 min resting-state frontal EEG signals.

**FIGURE 2:**
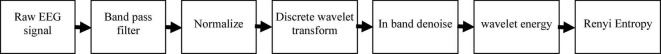
EEG processing flow diagram.

As shown in Figure 2, the data processing procedure contains six steps. The following parts of this section describe each of the processing steps.

#### Preprocessing

The recorded raw EEG signal was first filtered using a 3–30 Hz bandpass filter. The data were normalized through z-score normalization to remove variation introduced between assessments. The pre-processed EEG signals were calculated in 8-s windows with a 50% overlap between successive windows (Kar et al., 2010).

#### Discrete Wavelet Transform

We used discrete wavelet transform (DWT) to analyze the EEG signal. DWT analyses signals in different frequency bands with varying resolutions by decomposing them into coarse approximations and detailed information.

In the discrete domain, any finite energy time signal can be decomposed and expressed in scaled and shifted versions of a mother wavelet ψ(*x*) and a corresponding scaling function ϕ(*x*). The scaled and shifted version of the mother wavelet is defined by:


(1) $$\psi_{j,k}\left(x\right)=2^{\frac{j}{2}}\psi \left(2^{j}x-k\right)$$

The EEG signal *f*(*x*) can be described as the following equation:


(2) $$f\left(x\right)=\frac{1}{\sqrt{M}}\sum_{k}W_{\phi }\left[j_{0},k\right]\phi_{j_{0},k}\left[n\right]+\frac{1}{\sqrt{M}}\sum_{j=j_{0}}^{\infty }\sum_{k}W_{\psi }\left[j,k\right]\psi_{j,k}\left[n\right]$$

*W*_ϕ_ and *W*_ψ_ are the approximation coefficient and detail coefficient, respectively.

In this study, we used the Daubechies wavelet of order four (db4) because of its efficiency. The number of decomposition levels was set to four. The detail component from level one to level four is approximately corresponding to the β, α, θ, and δ band of the EEG signal.

The wavelet coefficient of the corresponding mother wavelet becomes higher when the signal has noise. The threshold *T_j_* can be computed as:


(3) $$T_{j}=mean\left(w_{j}\right)+2\times std\left(w_{j}\right)$$

*w_j_* is the wavelet coefficient at the *j*th level of decomposition, mean (.) and std (.) are the functions of computing mean and standard deviation. In-band filtering can reduce the absolute value of coefficient above the threshold by half (Euginia, 2005).

#### Entropy

To calculate Wavelet Entropy, wavelet energy *E_j_* of a signal is determined at each scale j as follows:


(4) $$E_{j}=\sum_{k=1}^{L_{j}}{\left[w_{j}\left(k\right)\right]}^{2}$$

Where, *L_j_* are the total number of coefficients at the *j*th level. Moreover, the relative wavelet energy at *j*th level can be calculated as:


(5) $$P_{j}=\frac{E_{j}}{\sum_{j}E_{j}}$$

∑_*j*_ *E*_*j*_ is the total energy over all levels.

The wavelet Rényi entropy is defined as


(6) $$RE=\frac{1}{1-q}\log\left[\sum_{j}p_{j}^{q}\right]$$

Where, *q* is the entopic index, it is a real number. The parameter *q* confers generality to this information measure. In this study, we have used *q* = 2 to calculate the 2nd order Rényi entropy.

### Statistical Analysis

Data analysis was performed using SPSS 21 (IBM Corp, Armonk, NY, United States). The differences between SSS score, oddball reaction time, and EEG wavelet Rényi entropy were analyzed using repeated measures analysis of variance (ANOVA). Time-of-day was included as a factor to determine whether the participants’ mental fatigue cognition and mental fatigue status changed with the continued competition. Based on the results of Mauchly’s test of sphericity, Greenhouse-Geisser was applied to all repeated measures ANOVA effects. *Post-hoc* comparisons (Least Significant Difference, LSD) were used to compare every two tests, a significance level of *P* < 0.05 was used for all statistical analyses.

The correlation coefficient between EEG wavelet Rényi entropy, oddball RT, SSS score, and pre-assessment sleep time was calculated using Person correlation.

## Results

### Comparisons Among Four Channels

All the variables presented displayed normal distribution (Shapiro-Wilk test), and the variances were homogeneous (Levene’s test). Using two-way (4 channels × 6 timepoints) repeated measures ANOVA to analysis four EEG channels in six timepoints; two-way (2 stimuli × 6 timepoints) repeated measures ANOVA analysis was used to analyze the changes of visual and audio oddball task in six timepoints. One-way repeated measures ANOVA analysis was utilized to analyze the difference of self-rating scores among six timepoints. We found that there is a significant difference between assessments day 1 to 5 (*F*_2.496,72.392_ = 21.491, *P* < 0.001). There were no significant differences between the four prefrontal EEG electrode positions (*F*_7.489,72.392_ = 0.263, *P* = 0.971). Therefore, the EEG signal at Fp1h has been used to illustrate the following results, the values of the other three channels can be found in the Supplementary.

### Comparisons Among Six Timepoints

**FIGURE 3:**
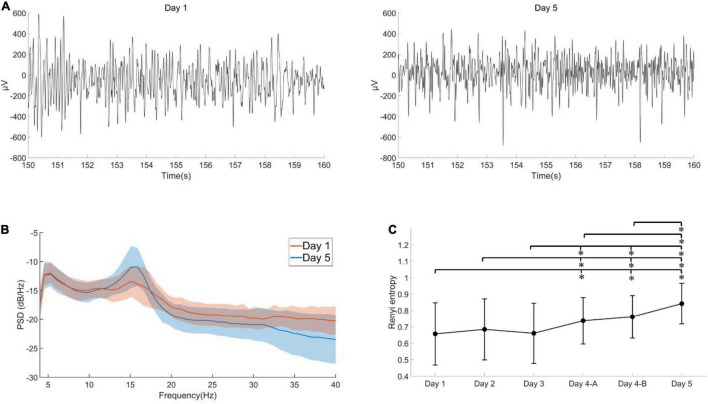
The analyzed results of PFC EEG. **(A)** 10 s filtered EEG data from Day 1 and 5. **(B)** The average power spectral density (PSD) of Day 1 (blue) and Day 5 (orange), the shade indicates the standard deviation of the PSD. The PSD value on Day 5 in the 13–18 Hz band is lower than that of Day 1. **(C)** The Rényi entropy of EEG over six assessment sessions. The Rényi entropy significantly increased over six sessions (*p* < 0.05, ANOVA), and it was significantly higher in the last session than in the first day. Day 4-A was the first assessment in day four, day 4-B was the second assessment in day four.

Figure 3A shows one participant’s 10 s filtered EEG data from Day 1 and 5. From Figure 3B, we can observe that the value of average power spectral density on Day 5 in the 13–18 Hz band is lower than that of Day 1. Through the Rényi entropy of EEG over six assessment sessions, we found no significant differences (*F*_1.19,33.31_ = 0.66, *P* = 0.448, repeated measures ANOVA) among first 3 days. As shown in Figure 3C, the change between days 1 – 3 is not apparent; however, day 4-A has a significant increase compared with days 1, 2, and 3 (*p* = 0.008, *p* = 0.044, and *p* < 0.0001). We found significant differences among the last three assessments through one-way repeated measures ANOVA (*F*_2,64_ = 22.818, *P* < 0.001). Relative to day 4-A and 4-B, day 5 demonstrated a significant gain, as Figure 3C shows. Assessments in days 4-A, 4-B, and 5 of the second phase were significantly different from days 1, 2, and 3 of the first phase; this is illustrated in Figure 3C, days 1 – 5, where mean values are gradually increasing. Table 1 shows the descriptive statistics of Rényi entropy, oddball reaction time, SSS score, and sleep duration across six assessments.

**TABLE 1:** Descriptive statistics of Rényi entropy, oddball reaction time, SSS score, and sleep duration across six assessments.

	**Day 1**	**Day 2**	**Day 3**	**Day 4-A**	**Day 4-B**	**Day 5**
**Fp1h Rényi entropy**						
Mean	0.657	0.684	0.661	0.737	0.761	0.841
SD	0.190	0.186	0.184	0.142	0.129	0.124
**Reaction time of visual (second)**						
Mean	0.338	0.334	0.360	0.351	0.366	0.404
SD	0.035	0.034	0.074	0.068	0.101	0.122
**Reaction time of audio (second)**						
Mean	0.328	0.320	0.331	0.353	0.374	0.398
SD	0.034	0.032	0.052	0.076	0.121	0.128
**SSS score**						
Mean	2.333	1.778	2.444	2.556	3.222	4.222
SD	0.707	0.667	0.882	0.882	1.302	1.716
**Sleep duration (minute)**						
Mean	375.5	419.4	243.	232.2	15.5	2.2
SD	64.3	34.1	139.3	69.4	39.7	6.6

**FIGURE 4:**
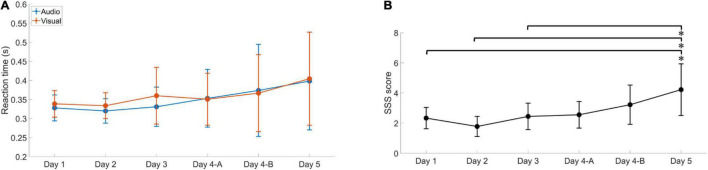
The reaction time for the oddball task and self-rated fatigue for the SSS over six assessment sessions. Error bars indicate the standard deviation. **(A)** The reaction time for the audio stimulation (blue) and visual stimulation (orange) increased as the competition progressed, and the bars indicate the standard deviation. **(B)** The self-rated fatigue increased as the competition progressed, and Day 5 is significantly different from the first three assessments. Day 4-A was the first assessment in day four, day 4-B was the second assessment in day four.

There was a significant difference between the six self-rating scores (*P* = 0.018), with day 5 having significantly increased levels of mental fatigue relative to the other assessments. The mean value of participants’ self-rated fatigue was increased through the assessments, and the highest score was slightly higher than four, as shown in Figure 4B. However, some individuals score barely changed during the whole experiment.

There was no significant difference between performances of the oddball task, though a trend toward significance can be seen (*P* = 0.066). From the *post-hoc* comparisons, we can observe that the score from day 5 is significantly different from days 2, 3, and 4-A. Figure 4A shows the average reaction times of the participants for the six oddball tasks. We can observe a gradual increase in reaction time from day 2 to 5. The decrease in measured reaction time from the first to the second time could be caused by the participants’ lack of proficiency in the assessment.

Sleep duration before the assessment decreased as the competition progressed. Correlation analysis showed that the SSS score and Rényi were negatively correlated with sleep time (rho = −0.486, *P* < 0.001, rho = −0.442, and *P* < 0.001). The audio and visual oddball reaction time did not correlate with sleep time (rho = −0.015, *p* = 0.913, rho = −0.228, and *P* = 0.100).

## Discussion

This study focused on objectively measuring mental fatigue changes during a long-term high-level stress competition using EEG-based marker, wavelet Rényi entropy and verified our claim by analyzing participants’ PFC EEG, self-reported scales, and the oddball task’s reaction time measured during a five-day real-world intellectually challenging competition.

The evidence supports our hypothesis that mental fatigue conditions become more severe as the competition progresses. The last assessment of mental fatigue is significantly different from the previous five assessments for all three measurement instruments. Also, the wavelet Rényi entropy of PFC EEG is a more sensitive indicator of mental fatigue than self-reporting and reaction time during the oddball task. These results suggest that mental fatigue will accumulate during long-term high-level stress conditions, and PFC activity analyses can identify it.

**FIGURE 5:**
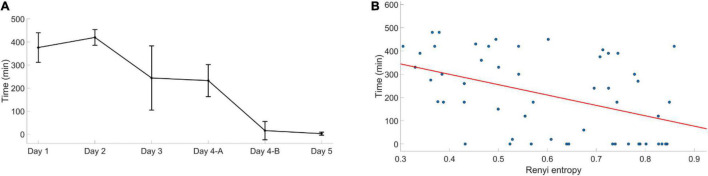
Sleep time changes during the competition. **(A)** The total sleep time before each assessment. Error bars represent standard deviation. Day 4-A was the first assessment in day four, day 4-B was the second assessment in day four. **(B)** The EEG Rényi entropy and sleep time correlation analysis.

The trend of the mental fatigue curve is gradually increasing, which means the mental fatigue conditions became more severe as the competition progressed. This increasing rate was different in two phases. In the first phase, the index fluctuated, and it did not show a significant difference between each assessment. This phenomenon may be related to the sleep recovery effect on mental fatigue (Belenky et al., 2003; Mantua et al., 2019). Moreover, the participant’s desire to win the competition, and subsequently the prize, is a short-term reward, and a study found that short-term reward make the body spend more energy to achieve it. In that case, working overtime will not lead to mental fatigue (Boksem and Tops, 2008). From Figure 5, we also can see that even the sleep duration had reduced, the fatigue state did not change much. However, even though sleep duration only decreased slightly when examining the second phase, the mental fatigue increased significantly.

When the contest entered the final day and the second phase of our assessment, we found that compared to phase one, the increase became larger for each measurement, especially for EEG wavelet Rényi entropy analysis. Such a tendency is a characteristic of fatigue accumulation (Borghini et al., 2014; Dohrmann et al., 2019). The alternation in EEG has been used to assess mental fatigue in the short term (Li et al., 2017), so this kind of change could be extended as a threshold value for long-term mental fatigue monitoring.

The three instruments used in this study showed consistency in the trend of changes in mental fatigue measurement. However, the SSS self-report was not sensitive enough to reflect the actual mental fatigue conditions. In our case, two participants’ self-ratings were inconformity with their reality. From the average SSS score shown in Figure 4B, we can see even on the last assessment, some participants under pressure from the competition and sleep-deprived still rated themselves feeling “a little foggy; not at peak; let down.” These results support that participants with severe mental fatigue have weak cognitive performance (Mantua et al., 2020), and the description of SSS items can be misinterpreted by participants (Horne, 2010).

The results showed no significant difference in reaction time for both the visual and audio oddball tasks in a repeated measures ANOVA. However, the results did approach the brink of significance (*P* = 0.066), and on *post-hoc* analyses, we observed that the assessment on day 5 is significantly different from days 2, 3, and 4-B. These results suggest that the oddball task reaction time is not sensitive to the mental fatigue assessment. This result is in accordance with the results of SYLVIA (Loh et al., 2004), where the reaction time during a cognitive task is used to evaluate fatigue needs to reach a certain duration, such as 10 min for the best result of PVT and 27 min for the oddball task used by Luis (Ciria et al., 2017) or fatigue detection. Since the assessments were administered during a real competition, we had to keep time for data collection from the participants as short as possible and compressed the response time of the cognitive task into two 4-min assessments, following the approach of Mathias (Basner et al., 2011). The results show that using the reaction time of the oddball task as a metric only showed significance in extreme conditions like sleep deprivation, the wavelet Rényi entropy analysis of PFC EEG demonstrated the same trend of values changes with better discrimination.

In light of these findings, our results may point to that results from SSS scores are susceptible to subjective bias, and the RT of the 4 min oddball task was not sensitive enough to long periods of mental fatigue. The poor performance of the oddball task in this study could be due to the small sample size; additionally, the lack of diversity makes it difficult to generalize our findings to a larger population. It is also worth noting that the short-term oddball task is not ideal for measuring mental fatigue in real-world scenarios. In this experiment, the PFC activity was more objective and effective in assessing accumulated mental fatigue.

Previous studies have used EEG to quantify stress states and have found EEG-related markers that can objectively and effectively assess stress (Peng et al., 2012; Minguillon et al., 2016; Keshmiri, 2021). The present study extended their thoughts to the case of mental fatigue under stress conditions. Moreover, this study supports that EEG wavelet Rényi entropy can be a quantitative marker that reflects different levels of mental fatigue under high-level stress conditions. Hence, EEG wavelet Rényi entropy would be expected to apply in future studies to measure mental fatigue.

The sample size is relatively small, which may have limited our ability to detect certain relationships in larger populations, such as the EEG wavelet Rényi entropy assessment results and reaction time change in the oddball task. Future studies need to extend the sample size to examine and confirm the EEG wavelet Rényi entropy and oddball assessment results. Furthermore, stress can affect the brain’s state (Hermans et al., 2011; Keshmiri, 2020) and how the stress eventually leads to mental fatigue need further investigation.

## Conclusion

This study investigated mental fatigue changes during a 5-day intellectually challenging competition and found that mental fatigue accumulates during long-term high-level stress situations. We further demonstrated that the PFC EEG wavelet Rényi entropy is a more sensitive instrument than self-reporting and oddball reaction times to measure mental fatigue accumulation. These results may help improve research on long-term mental fatigue.

## Data Availability Statement

The raw data supporting the conclusions of this article will be made available by the authors, without undue reservation.

## Ethics Statement

The studies involving human participants were reviewed and approved by Ethics Committee of the Institute of Science and Technology for Brain-inspired Intelligence. The patients/participants provided their written informed consent to participate in this study.

## Author Contributions

CL and HZ conceived, planned, and carried out the experiments. HZ performed the analytic calculations and statistical analysis, and wrote the first draft of the manuscript. JW, SW, and XG verified the analytical methods. CL and SW helped supervise the project. All authors contributed to manuscript revision, read, and approved the submitted version.

## Conflict of Interest

The authors declare that the research was conducted in the absence of any commercial or financial relationships that could be construed as a potential conflict of interest.

## Publisher’s Note

All claims expressed in this article are solely those of the authors and do not necessarily represent those of their affiliated organizations, or those of the publisher, the editors and the reviewers. Any product that may be evaluated in this article, or claim that may be made by its manufacturer, is not guaranteed or endorsed by the publisher.
