# Supplementary material for: Objective Assessments of Mental Fatigue During a Continuous Long-Term Stress Condition
Source: Front Hum Neurosci. 2021 Nov 10;15:733426. doi: 10.3389/fnhum.2021.733426 (PMC8631328; doi:10.3389/fnhum.2021.733426)
Supplement: Supplementary file 1 [file Data_Sheet_1.docx]

Supplementary Material


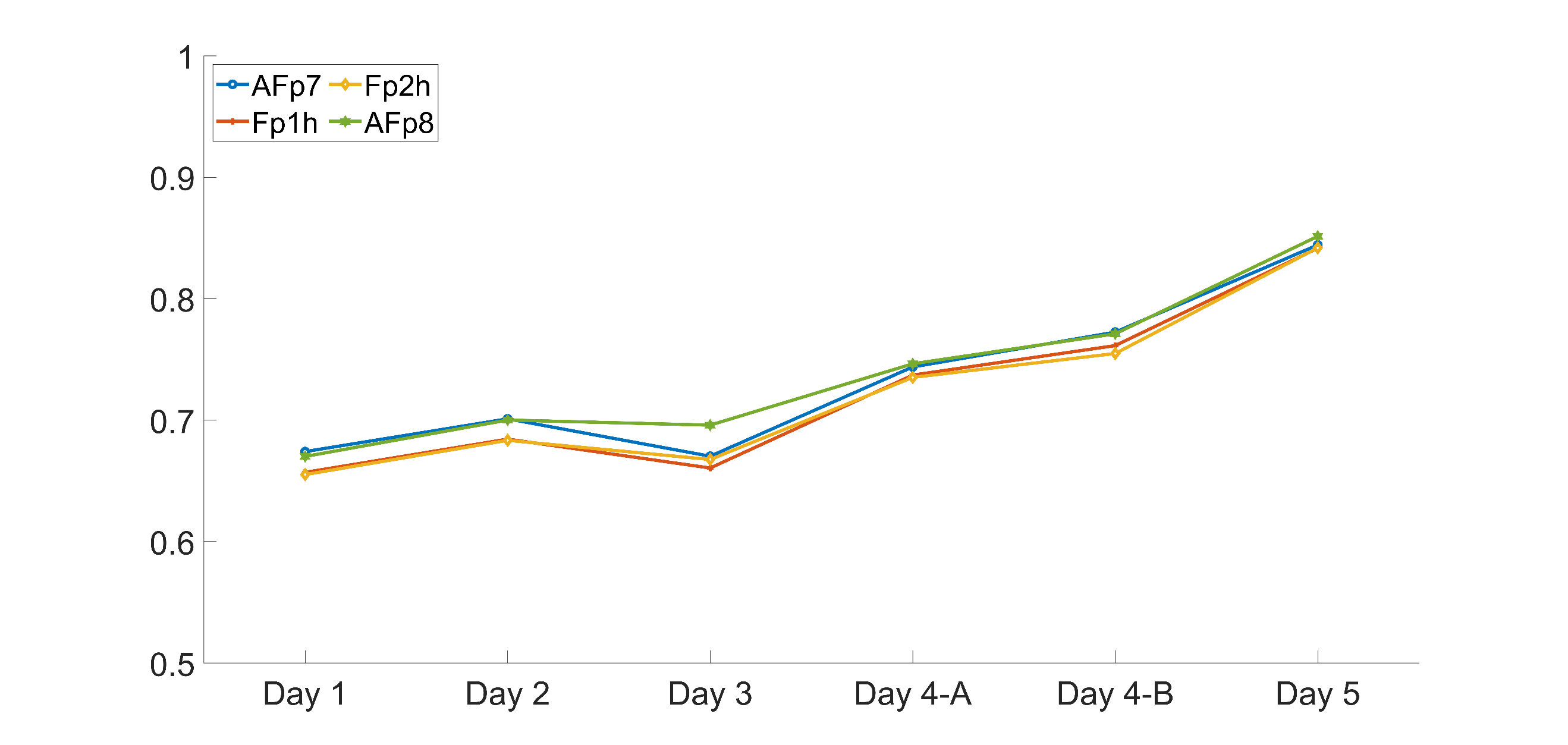


**Supplementary Figure 1.** The mean value of EEG wavelet Rényi entropy in six assessments.

Table 1 The mean value of EEG wavelet Rényi entropy

|  | Day 1 | Day 2 | Day 3 | Day 4-A | Day 4-B | Day 5 |
| --- | --- | --- | --- | --- | --- | --- |
| AFp7 | 0.6741 | 0.7011 | 0.6703 | 0.7438 | 0.7725 | 0.8443 |
| Fp1h | 0.6570 | 0.6844 | 0.6606 | 0.7371 | 0.7614 | 0.8416 |
| Fp2h | 0.6552 | 0.6834 | 0.6676 | 0.7352 | 0.7549 | 0.8419 |
| AFp8 | 0.6701 | 0.7001 | 0.6959 | 0.7464 | 0.7711 | 0.8514 |
